# Molecular analysis of mucopolysaccharidosis type I in Tunisia: identification of novel mutation and eight Novel polymorphisms

**DOI:** 10.1186/1746-1596-6-39

**Published:** 2011-04-26

**Authors:** Latifa Chkioua, Souhir Khedhiri, Asma Kassab, Amina Bibi, Salima Ferchichi, Roseline Froissart, Christine Vianey-Saban, Sandrine Laradi, Abdelhedi Miled

**Affiliations:** 1 Biochemistry laboratory Farhat Hached Hospital, Street Doctor Moreau, 4000 Sousse - Tunisia; 2 Biology Molecular laboratory University of Pharmacy 5000 Monastir - Tunisia; 3 Biology Molecular laboratory Child Hospital Tunis-Tunisia; 4 Hereditary service of metabolic diseases and neonatal screening. Center of biology and pathology. 69677 BRON CEDEX France

**Keywords:** Mucopolysaccharidosis type I, IDUA gene, mutations, polymorphisms, Tunisian patients

## Abstract

Mucopolysaccharidosis type I (MPS I) is an autosomal recessive lysosomal storage disorder caused by a genetic defect in alpha-L-iduronidase (IDUA) which is involved in the degradation of dermatan and heparan sulfates. The disease has severe and milder phenotypic subtypes. The aim of this study was the detection of mutations in the IDUA gene from 12 additional MPS I patients with various clinical phenotypes (severe, 8 cases; intermediate, 3 cases; mild, 1 case).

**Patients and methods:**

In this study, the IDUA mutations in eight unrelated Tunisian families were performed by amplifying and sequencing the IDUA exons and intron-exon jonctions.

**Results:**

Five IDUA mutations were detected: one is the L578Q, a novel mutation found, in milder patient. The others were the previously described: P533R, Y581X, F602X and R628X that produce a severe and intermediate phenotype. In addition, eighteen variants, including eight previously unreported polymorphisms (IVS6+21c > a, IVS7+79c > t, IVS7-45 g > c, IVS9+36t > c, IVS10+140c > a, IVS11+33c > t, IVS12+13c > t and IVS12-31c > g), were detected.

**Conclusion:**

This paper, showed a heterogeneous pattern of mutations and polymorphisms among Tunisian patients.

## Background

Mucopolysaccharidosis type I (MPS I) is an autosomal recessive lysosomal storage disorder that results from a deficiency in the alpha-L-iduronidase (IDUA; E.C.3.2.1.76). This glycosidase is involved in the degradation of dermatan and heparan sulfates. IDUA deficiency causes a progressive accumulation of undegraded mucopolysaccharides leading to characterized clinical phenotypes [1]. MPS I has three major clinical phenotypes, ranging from the severe Hurler form to the Scheie phenotype:

The severe phenotype (Hurler syndrome, MPS IH) involves mental retardation, skeletal of deformities, stiff joints, hepatosplenomegaly, corneal clouding, and a shortened life expectancy. The attenuated phenotype (Scheie syndrome, MPS IS) is characterized by mild skeletal deformities, stiff joints, corneal clouding and a long life span without mental retardation. The intermediate phenotype (Hurler / Scheie syndrome, MPS IH/S) involves skeletal deformities, severe organomegaly, usually limited bone involvement and a variable life span with neurological involvement. The wide spectrum of clinical severity in MPS I suggests the presence of allelic heterogeneity.

The IDUA gene is located on chromosome 4p16.3. It contains 14 exons spanning 19 kb. It is transcribed into 2.3 kb cDNA, which encodes a 653 amino acids protein [2,3]. Human Gene Mutation Database described more than 100 mutations and 32 polymorphisms (http://www.hgmd.org). Previous mutations including missense and large deletion have been found in patients with the severe phenotype. The others gender such as nonsense, insertion-deletion and splice site have been identified in both severe and mild phenotypes.

In Tunisia, first cousin inbreeding is the most represented with an estimated incidence about 32% [4]. Furthermore, Tunisian population is characterized by a heterogeneous genotype resulting from social assets created by invasions and migrations. For example, it exists a large mutations spectrum of hemoglobinopathies such as beta-thalassemia identified as a result of rare alleles selection [5]. Thus, screening IDUA mutation in MPS I patients is essential to identify the specific mutation of our country, to detect heterozygote and prenatal diagnosis.

In this study, we investigated IDUA mutation in MPS I Tunisian families based on a complete scan of IDUA gene.

## Patients and methods

### Patients

**Figure 1:**
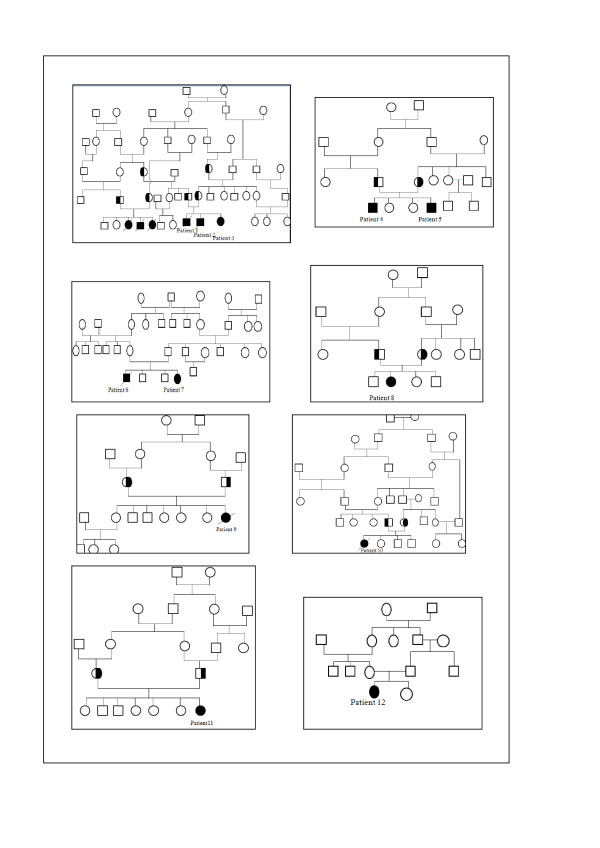
**Pedigrees of Tunisian MPS I families**.

We studied the mutations in 8 unrelated families (Table four). All patients were of central and southern Tunisia origin. The patients were all offspring of consanguineous mating (Figure 1). A total of 24 alleles were investigated. Diagnoses were confirmed by demonstration of deficiency IDUA activity in leukocytes. The clinical evaluation of the patients included complete medical history, physical examination routine haematological and biochemical tests and measurement of leukocytes IDUA activities. Patients were assigned to one of three clinical phenotypes according to prior descriptions: 8 severe, 3 intermediate and 1 mild.

#### Patients of family 1

This family gave birth for 6 patients. Three were died before our investigation at on age of one year, one year and 3 months and 12 years. The other three patients: the first boy was diagnosed at the age of three months when he was operated for inguinal hernia. Coarse facial feature were noted at an age of eight months. At the age of 5 years he developed deafness and chronic otitis. Then, he deceased as a result of pulmonary and cardiac deficiency. His brother developed similar coarse facial features at the age of fifteen months. He showed severe developmental delay, hydrocephaly, deafness and inguinal hernia. He deceased at the age of sixteen months.

These two patients presented the classical severe phenotype of Hurler disease accentuated in the second boy. Their sister was diagnosed at the age of nine months. She developed facial dysmorphism, skeletal dysplasia. This family has beneficiated a prenatal diagnosis.

#### Patients of family 2

The two boys are developed the same severe phenotype: facial dysmorphisms, severe skeletal abnormality, cornea clouding, umbilical hernia and severe mental retardation.

#### Patients of family 3

The two patients were a boy of sixteen years old and his sister of thirteen years old. The boy was characterized by corneal clouding, moderate skeletal abnormalities, moderate joint stiffness and valgus deformity. His sister had very moderate facial dysmorphisms. She was fortuitously identified at the age of five years old during our survey. The clinical features of these two patients were markedly more moderate than the other Hurler affected children, demonstrating the Hurler / Scheie phenotype. The patient deceased at the age of twenty five years old; however his sister is still survived.

#### Patient of family 4

The patient had gradually developed a facial dysmorphism, moderate skeletal abnormalities and short status. He presented the Hurler / Scheie phenotype. Despite their phenotype, he carried on a normal school education.

#### Patient of family 5

The girl was diagnosed at the age of one year. She presented progressively a severe mental retardation, short status, umbilical hernia, spacing and shape teeth, enlarged tonsils, facial dysmorphism, corneal clouding and dysostosis multiplex. She was a black girl with pigmented skin. She deceased at the age of five years old. She presented the severe Hurler phenotype.

#### Patients of family 6

The girl was diagnosed at the age of five years. She had developed facial dysmorphism, severe mental retardation, and hepatomegaly. She deceased at eight years old as a result of pulmonary and cardiac deficiency.

#### Patient of family 7

She was diagnosed at the age of one year. She had developed gradually a facial dysmorphism, scaphocephaly, umbilical hernia. Her intelligence was normal.

#### Patients of family 8

The girl was diagnosed at the age of nine months. She presented facial dysmorphism, umbilical hernia and corneal clouding. At the age of four years she had developed severe skeletal abnormalities, short status and severe mental retardation. She presented the severe Hurler phenotype. She deceased at five years old.

### IDUA activity

IDUA activity was carried out by colorimetric enzyme assay using phenyl -α-L-iduronide as a substrate in sonicated fresh leukocyte pallets. Results were expressed by μKat/kg of leukocyte protein. To the method validation we measured the β-glucuronidase activity using the 4- methylumbelliferyl β-D-glucuronide. Total protein was established according Hartree method [6].

### IDUA molecular analysis

Genomic DNA was extracted from peripheral blood leukocytes using a standard phenol/chloroforme procedure [7]. Each of 14 exons and flanking intron-exon junctions was amplified as described previously [3].

Polymerase chain reaction (PCR) was carried out in 50 μl total volume containing 50 ng genomic DNA, 0.2 mmol/L dNTP, 0.8 pmol of each primer, 0.08 mmol/L MgCl_2_. We used two types of Taq polymerase: 1.5 U Hot Start Qiagen Taq (to amplify 4, 5, 6, 7, 8, 9 and 13 IDUA exons) and 2 U Hot Start Qiagen Taq (to amplify 2 and 3 IDUA exons) and 1.5 U QBiogen Taq (to amplify 1, 10 and 14 IDUA exons).

Cycling conditions were: 95°C for 3 min, followed by 40 cycles for 30s at 95°C, 30s at 58-65°C, 30s at 72°C and final extension at 72°C for 7 min.

Successfully amplified PCR products were purified from excess primers and dNTP using plaques Millipore [LSKSO 9624 Millipores]. Direct sequencing was performed with a Big Dye termination Kit (Applied Biosystems) in both forward and reverse directions with the same PCR primers and analyzed on an ABI Prism 3130 ×l capillary Array Sequencer (Applied Biosystem).

## Results

### Clinical features and IDUA activity

The clinical features of each patient and leukocyte IDUA activities are presented in Table 1. IDUA activities ranged from 0 to 0.05 μKat/kg of protein. (Normal values 1.7 →3.5 μKat/kg of protein).

**Table 1:** Clinical characteristics of Tunisian MPS I patients

FAMILLES	I			II		III		IV	V	VI	VII	VIII
Patients	1	2	3	4	5	6	7	8	9	10	11	12

Age	5 years 6 months (died)	1 year 3 month (died)	9 months	9 years (died)	10 years	25 years(died)	13 years	10 years	5 years (died)	8 years (died)	6 years	5 years

Age of onset	2 months	1 month half	9 months	7 months	8 months	9 months	5 years	1 year 6 months	1 year	5 years	1 year	9 months

Sex	Male	Male	Female	Male	Male	Male	Female	Male	Female	Female	Female	Female

Height (cm)	103 (-1,5 DS)	71 (+1DS)	51(+1DS)	107 (-3 DS)	118 (-3 DS)	120 (-3 DS)	110 (-3 DS)	115 (-3 DS)	102 (-3DS)	107 (-3DS)	120(3DS)	120(3DS)
Weight (Kg)	13 (-2 DS)	12 (+2 DS)	7(+1DS)	19 (-1 DS)	18 (- 3 DS)	20 (-3DS)	15 (- 1 DS)	19 (-1 DS)	18 (-1 DS)	19 (-1DS)	20(-3DS)	18(-1DS)

IDUA activity (μKat/kg)	0,00	0,00	0,00	0,018	0,018	0,030	nd	0,050	0,020	nd	0.09	0.00

Nd: non determined

### IDUA mutations analysis

We analyzed the IDUA gene of 12 MPS I patients from Tunisia. The affected probands in the eight families proved the presence of eight cases of Hurler phenotype, two cases of Huler / Scheie phenotype and one case of Scheie phenotype (Table 2). By direct sequencing of the IDUA gene, we found four previously reported mutations: F602X, P533R, Y581X and R628X and one novel mutation: L578Q. All mutations and polymorphisms were confirmed in the parental DNA.

**Table 2:** Genotypes Characteristics of MPS I Tunisians patients

families	patients	Parental consanguinity	MPS I phenotype	Mutations	Polymorphisms
I	1	3rd cousins	severe		A8A, A20A, H33Q, R105Q, A314A
	2			F602X/F602X	
	3				

II	4	1st cousins	severe	P533R/P533R	Not tested
	5				

III	6	1st cousins	intermediate	P533R/P533R	A8A, A20A, H33Q, R105Q, A314A
	7				

IV	8	3rd cousins	intermediate	P533R/P533R	Not tested

V	9	1st cousins	severe	R628X/R628X	A8A, A20A, H33Q, R105Q, A314A, N181N, T410T, V554I, IVS6+21c > a, IVS7+79c > t, IVS7-45 g > c, IVS9+36t > c, IVS10+140c > a, IVS11+33c > t, IVS12+13c > t, IVS12-31c > g

VI	10	1st cousins	severe	P533R/P533R	A8A, A20A, H33Q, R105Q, A314A, N181N, T410T, V554I, IVS6+21c > a, IVS7+79c > t, IVS7-45 g > c, IVS9+36t > c, IVS10+140c > a, IVS11+33c > t, IVS12+13c > t, IVS12-31c > g

VII	11	2 nd cousins	mild	L578Q/P533R	A8A, A20A, H33Q, R489R

VIII	12	2nd cousins	severe	Y581X/Y581X	H33Q

The probands of family one were homozygous for F602X mutation. The probands of families tow, three, four and six were homozygous for P533R mutation. The proband of family five was homozygous for R628X mutation. The proband of family seven was heteroallelic for the novel mutation L578Q and P533R mutation. The proband of family eight was homozygous for the Y581X mutation.

Furthermore, we identified eighteen sequence variants, including eight previously novel polymorphisms. The reported polymorphisms were: A8A, A20A, H33Q, R105Q, A314A, N181N, T410T, V454I, R489R and IVS10+53 g > t. The unreported polymorphisms were: IVS6+21c > a, IVS7+79c > t, IVS7-45 g > c, IVS9+36t > c, IVS10+140c > a, IVS11+33c > t, IVS12+13c > t, IVS12-31c > g (Table 3).

**Table 3:** Table 3 polymorphisms characteristics and position in MPS I Tunisians patient

Nucleotide change	cDNA position	gDNA position	Exon/intron	codon/nucleotide change	Restriction enzyme	References
GCC > GCA	112	606	Exon 1	A8A	(+ ) Eco47III	[13]

GCG > GCA	148	642	Exon 1	A20A		[11]

CAG > CAT	187	681	Exon 1	Q33H	(+) Nsp 7524I	[13]

CGG > CAG	402	441	Exon 3	R105Q	(+) AluNI	[2]

AAT > AAC	631	1332	Exon 5	N181N		[25]

GCG > GCC	1030	1946	Exon 7	A314A		[13]

ACC > ACG	1318	2587	Exon 9	T410T		[13]

GTC > ATC	1447	2716	Exon 9	V454I		[13]

CGC > TGC	1553	2911	Exon 10	R489R		[25]

ccg > cag	887+21	1836	Intron 6	IVS6+21c > a	(-) AciI	^a^

acg > atg	1060+79	2055	Intron 7	IVS7+79c > t		^a^

agc > acc	1061-45	2039	Intron 7	IVS7-45 g > c		^a^

ctg > ccg	1487+36	2756	Intron 9	IVS9+36t > c	(+) NciI	^a^

ggg > gtg	1612+53	3052	Intron 10	IVS10+53 g > t		[25]

gcg > gag	1612+140	3165	Intron 11	IVS10+140c > a		^a^

ccc > ctc	1720+33	3318	Intron 11	IVS11+33c > t	(-) MspI	^a^

gcc > gtc	1817+12	3420	Intron 12	IVS12+13c > t	(-) Sau96I	^a^

acc > agc	1819-32	3741	Intron 12	IVS12-31c > g	(+) BseYI	^a^

^a^: Novel sequence changes described in our patients.

## Discussion

MPS I is the most common subtype of mucopolysaccharidosis with an estimated of about 1.7 in 100. 000 live births for the severe and mild forms [8]. The incidence of MPS I in Tunisia is also high, estimated at 0.63 in 100.000 live births [9]. MPS I patients show a wide spectrum of clinical phenotype ranging from the severe Hurler to the attenuated Scheie form [10]. Currently, over 100 mutations have been reported in patients with the MPS subtypes (Human Gene Mutation Database; http://www.hgmd.org).

**Figure 2:**
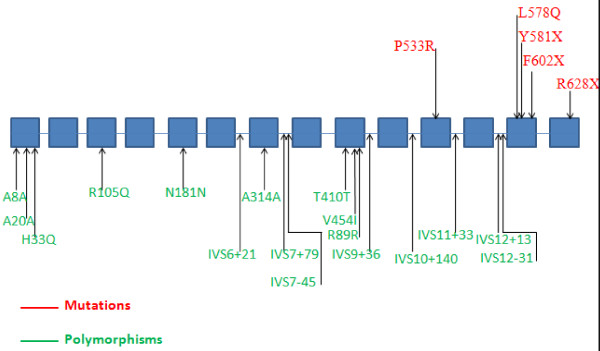
**Mutations and polymorphisms of IDUA gene**.

In our investigation, all patients were born from to consanguineous marriages between first, second and third cousins (Figure 1). The molecular analysis of the MPS I patients in eight Tunisian families, identified five mutations including a novel mutation: L578Q and four reported mutations: P533R, Y581X, F602X and R628X (Figure 2). Of note, the common mutations, Q70X and W402X in European populations were not found [11].

Patients 1, 2 and 3 from family one were homozygous for the F602X mutation. This predicts the synthesis of a premature chain termination of the IDUA glycopeptide. These patients have undetectable IDUA activity. F602X mutation was firstly reported in Tunisian patient as deletion-insertion complex in exon 13 of the IDUA gene [12]. Besides, our study confirmed the absence of F602X mutation in 50 DNAs of our normal population.

Patients 4, 5, 6, 7, 8 and 10 from families two, three, four and six were homozygous for the P533R mutation in exon 11 of IDUA gene. The P533R missens mutation was first described in a group of 73 MPS I patient (3%) [13]. Alif et al 2002 [14] reported a high frequency of this mutation in a group of 13 MPS I patients from Morocco (92%). This mutation has been identified also in 10 MPS I patients (62.5%) from Tunisia [15]. The P533R mutation has been identified in a group of 27 MPS I patients (11%) from Sicily [16] and not identified in 3 MPS I patients from Egypt [17].

Some studies of genetic and population background for different inherited diseases such as Niemann-Pick [18], metachromatic leukodystrophy [19], Gaucher disease [20] and mucopolysaccharidosis type I [21] succeed to distinguish the origin of frequent mutation. Haplotype analysis may distinguish whether a relatively common mutation is resulted from a founder effect or from recurrent mutations or multiple haplotypes. Thus for P533R origin estimation we think that haplotypes analysis will be determined for different MPS I populations.

The patient of family five was homozogous for the previously reported R628X mutation. This patient had the severe Hurler phenotype and she deceased at five years of age. We showed in a previous study that this patient was probably homozygous for this mutation. This finding is a confirmation of previous hypothesis [15].

The patient of family seven was heteroallelic for the novel L578Q mutation and the previous reported P533R mutation. This patient had the typical Scheie phenotype. The P533R mutation resulted in a non conservative substitution of a neutral proline for a basic arginine and associated with a low level of residual IDUA enzymatic activity in CHOK I cells [22]. The novel L578Q mutation presumably also causes a mild instability or conserves of function since the patient have the attenuated phenotype. She goes at school and she had a good note in scientific subjects. Our study showed that L578Q mutation is not found in 50 DNAs of normal population.

The patient of family eight was homozygous for the previously described Y581X mutation. This patient had the severe Hurler phenotype and she deceased at five years of age.

A large number of polymorphisms and non-pathogenic sequence variants have been described in the IDUA gene [23,22,24]. The effect of these sequence variants on the IDUA activity has not been clearly defined, especially when they are associated with specific mutations. However, it has been speculated through structural effects on IDUA gene that such polymorphisms may modify the patient clinical phenotype [25,26], particularly for patients who have missense mutations causing the Hurler / Scheie and Scheie phenotypes [11]. We found the same previously described polymorphisms associated to different phenotypes: Hurler and Hurler / Scheie form for patients of family one and three. Besides, the novel polymorphisms were associated to severe phenotype for patient of family five and six.

Mutations which permit some enzymatic function may be more susceptible to modulation by variables factors such as novel combinations of mutant alleles, attenuating polymorphisms, genetic background, and environmental factor (Tables 2, 4).

**Table 4:** Background of the MPS I families

Families	I	II	III	IV	V	VI	VII	VIII
relationship of parents	Parents are cousins	Parents are cousins	The parents have the same grand father with different wife	Parents are cousins	Parents are cousins	Parents are cousins	Parents are cousins	Parents are cousins

age of parents at birth	38 years (M) 40 years (F)	55 years (M) 65 years (F)	40 years (M) 49 years (F)	34 years (M) 40 years (F)	50 years (M) 62 years (F)	42 years (M) 50 years (F)	37 years (M) 44 years (F)	38 years (M) 46 years (F)

occupation	In a society	In a society	No occupation	No occupation	No occupation	No occupation	In a society	No occupation

specific conditions (environment (city/rural area))	Sfax: Industrial region	Gabes: Industrial region	Djerba: Land	Tunis: Industrial region	Mahdia: Touristic region	Nfidha: Industrial region	Tunis: Industrial region	Tunis: Industrial region

M: Mother

F: Father

Screening of polymorphisms in IDUA gene and determination of the haplotypes is necessary to search the origin of mutations within MPS I patients. Individuals resulting from a common ancestor are likely to have inherited both copies of the mutated gene and also in the haplotypes which will be transmitted to descent and thus rare genotypes are maintained [27].

Further studies in determination of haplotypes within MPS I patients might help in finding out whether these haplotypes are associated with the reported mutations and polymorphisms.

## Conclusion

In the present paper, eighteen IDUA sequence variants have been identified of MPS I Tunisian patients, including eight novel polymorphisms. The effect of noncoding and coding polymorphisms on IDUA expression in the MPS I patients from Tunisia is unclear leading to ambiguous correlation genotype / phenotype establishment. Further studies on large number of MPS I patients and normal population might help in finding out whether these novel and reported polymorphisms are associated with a specific phenotype.

## Abbreviations

MPS I: Mucopolysaccharidosis I; IDUA: Alpha L iduronidase; PCR: polymerase chain reaction.

## Competing interests

The authors declare that they have no competing interests.

## Consent

Written informed consent was obtained from the patient for publication of this case report and accompanying images. A copy of the written consent is available for review by the Editor-in-Chief of this journal.

## Authors' contributions

LC and SK have done all the work (PCR, sequencing...) in the laboratory. AK and AB have done the analysis of the results. RF and CVS interpret the results. S F, S L and A M have given final approval of the version to be published. All authors read and approved the final manuscript
